# New Strategies to Improve the Quality and Safety of Seafoods and the Efficient Utilization of Their By-Products

**DOI:** 10.3390/foods15020190

**Published:** 2026-01-06

**Authors:** Mingyu Yin, Xichang Wang

**Affiliations:** College of Food Science and Technology, Shanghai Ocean University, Shanghai 201306, China; myyin@shou.edu.cn

## 1. Introduction

As global economic development has significantly raised living standards, seafood has become an increasingly essential component of the human diet. Marine organisms supply high-quality protein, unsaturated fatty acids, vitamins, and trace elements, and are also rich in physiologically active compounds that support human health [1,2,3]. In an era when trends toward healthy and functional diets are increasingly prominent, seafood has attracted particular attention for its nutritional and health benefits. However, rising consumer demand has outpaced improvements in the sector; rapid industry expansion has been accompanied by uneven product quality, recurrent safety incidents and extensive by-product waste [4,5,6]. The development of innovative technologies and management strategies to enhance product quality, ensure safety, and promote high-value utilization of by-products has become an urgent focus of the marine food industry [7].

Seafood quality and safety remain principal challenges for the sustainable development of the seafood industry. The variable marine environment, together with the complex processes of harvesting, aquaculture, processing, storage, and transport, exposes seafood to multiple hazards, including microbial contamination, heavy metal accumulation, and persistent environmental pollutants [8,9]. In particular, lipid oxidation, protein denaturation, and changes in color and texture further compromise product quality and marketability during processing, storage, and transport [10,11]. Conventional methods, such as heat treatments, pickling, and drying, can prolong shelf life but often result in nutrient loss and flavor deterioration [12,13,14]. Consequently, current research increasingly focuses on innovative processing technologies that preserve nutritional and sensory quality while improving safety.

In recent years, novel non-thermal processing technologies have opened new avenues for enhancing the quality and safety of seafood products [12]. For example, high-pressure processing effectively inactivates microorganisms and endogenous enzymes while preserving nutrients [15]. Microwave-assisted processing and pulsed electric field treatment promote the extraction of bioactive compounds and improve tissue structure [16,17]. Cold plasma, notable for low-temperature sterilization, has been widely investigated for deodorization and oxidation control on seafood surfaces [18,19].

Efficient, high-value utilization of marine by-products is a key pathway toward the sustainable development of the marine food industry [7]. Seafood processing generates large quantities of by-products—fish bones, skin, scales, viscera, and shells—that are often discarded or otherwise used at low value. This practice squanders valuable marine biological resources and can cause environmental pollution [20,21]. In fact, these by-products are rich in high-quality proteins, collagen, polysaccharides, minerals, and lipids and can be valorized through advanced biotechnological or chemical processes to yield novel, value-added raw materials for the food, pharmaceutical, and cosmetic industries [22,23].

In recent years, green extraction and biotransformation technologies have emerged as focal areas of study. For example, enzymatic hydrolysis of fish skins and bones yields collagen peptides with antioxidant and anti-aging properties [24,25]. Supercritical CO_2_ extraction efficiently produces high-value products such as cod liver oil rich in ω-3 fatty acids [26,27]. Modified chitosan derived from shellfish by-products can be converted into high-performance biodegradable packaging films and sustained-release drug carriers [28,29]. Algal by-products likewise show promise as sources of food thickeners and functional ingredients [30]. The high-value utilization of seafood by-products thus enhances resource-use efficiency and aligns with circular-economy and environmental objectives. Establishing a circular system across the entire seafood industry and converting “by-products” into “recycled resources” is central to achieving green growth in the marine economy [22,31].

The improvement of seafood quality and the high-value utilization of by-products are not separate concerns; they must be advanced in concert through a systems-level perspective and a full-chain approach. Integrating novel processing technologies can both enhance the quality of primary products and ensure their safety. Concurrently, rational, high-value utilization of by-products extends industrial value chains and optimizes the industrial ecosystem. Interdisciplinary research is therefore increasingly important: coordinated innovation across food science, biochemistry, environmental engineering, materials science, and information technology will sustain long-term vitality in the marine food industry.

This Special Issue, “New Strategies to Improve the Quality and Safety of Seafoods and the Efficient Utilization of Their By-Products”, was conceived against that backdrop. It aims to bring together recent research from international academic and industrial communities on topics including seafood quality and safety enhancement, novel processing technologies, the extraction of functional components, and by-product valorization. By presenting innovative methods and leading scientific advances, the Issue intends to provide new evidence and practical applications for the global marine food sector and to help shift development from mere quantitative growth toward a balanced emphasis on quality improvement and sustainable use.

## 2. Key Contributions and Their Implications

This Special Issue aims to gather original research and review articles exploring innovative strategies for quality and safety assurance, microbial risk prevention and control, sustainable processing technologies, and the high-value utilization of seafood by-products. Particular emphasis is placed on the application of natural functional components, optimization of processing methods, elucidation of safety mechanisms, and efficient resource utilization. The nine contributions featured in this Issue collectively reflect the breadth of current research in the seafood sector, encompassing the modernization of traditional processing techniques, the adoption of emerging technologies, strategies for safety risk management, and the valorization of seafood by-products. The following section provides a concise overview of each paper, encouraging readers to explore the full articles for deeper understanding and inspiration.

The feature article of this Issue, authored by Liu et al. (Contribution 5), investigates the effects of steam treatment on the physicochemical properties and flavor characteristics of yellow pufferfish (Takifugu flavidus). The authors systematically examined the influence of different steaming durations on maturation, texture, color, and flavor, and established differentiated processing parameters for specific applications. Specifically, 9 min achieved optimal maturation; 10 min was suitable for industrial ready-to-eat production due to improved texture and color stability; and 15 min provided the richest flavor, making it ideal for home cooking. This study not only contributes to improving the food safety of pufferfish but also optimizes its quality under various processing scenarios, underscoring the central role of precision processing in balancing safety and sensory appeal. Moreover, it provides a replicable processing framework for the standardization of other high-risk seafood species.

From a precision-processing perspective, Li et al. (Contribution 8) further expanded the research scope of seafood dehydration by comparing four drying techniques—hot-air drying, vacuum hot-air drying, microwave drying, and vacuum freeze-drying (VFD)—applied to the adductor muscles of scallops (Patinopecten yessoensis). The results demonstrated that VFD most effectively preserved tissue integrity and reduced protein oxidation. VFD achieved a significantly higher rehydration rate (186.5%) and recovery rate (78%) than the other methods, confirming the advantages of gentle processing in maintaining both the nutritional and sensory attributes of seafood. This work extends the “processing–quality” paradigm highlighted in the lead article, offering further evidence of the value of precision control in seafood processing.

In advancing pickling process optimization, Ma et al. (Contribution 9) addressed key challenges in freshwater fish processing by comparing vacuum and atmospheric-pressure impregnation of grass carp fillets. Using low-field nuclear magnetic resonance (LF-NMR) and microstructural analysis, they demonstrated that a vacuum environment accelerates salt diffusion and promotes water redistribution, thereby increasing water-holding capacity, reducing lipid oxidation, and improving textural quality. Their findings provide a scientific basis for low-salt, health-oriented processing of grass carp and similar freshwater species, and further enrich the technological framework of precision seafood processing.

Effective control of safety risks forms the foundation of seafood processing. In Contribution 6, Wang et al. conducted phenotypic and genotypic characterization of two histamine-producing Morganella strains (GWT 902 and GWT 904) isolated from yellowfin tuna, addressing the hazard of histamine formation during cold-chain storage. The study delineated the low-temperature histamine-producing traits of a psychrotolerant strain (GWT 902) and elucidated the role of the histidine decarboxylase gene cluster (hdcT1, hdc, hdcT2, hisRS), thereby providing a scientific basis for risk-target identification in seafood cold-chain logistics and bridging a knowledge gap concerning psychrotrophic toxigenic Morganella in this field.

Complementing this work, Wang et al. (Contribution 3) examined the formation of harmful compounds during the frying of squid and found that flavonoids extracted from coconut exocarp more effectively inhibit the loss of polyunsaturated fatty acids and suppress the generation of toxic aldehydes than EDTA-2Na. They thus proposed a natural, safe strategy for processing-related risk control, opening a new avenue toward the development of healthier fried-seafood technologies.

A quality evaluation and traceability system is essential for processing standardization. Yu et al. (Contribution 1) developed a lipid-based quality assessment for female Eriocheir sinensis from three habitats in the lower Yangtze River. Their study identified the overall superiority of estuarine specimens and delineated habitat-dependent quality differences, thereby providing a scientific basis for seafood grading, traceability, and informed consumer choice, and strengthening the quality-assurance chain from origin to table.

The high-value utilization of seafood by-products is essential for sustainable industrial development. Huang and colleagues (Contribution 4) addressed the issue of 40% kelp (Laminaria japonica) scrap waste during processing by employing natural microbial community fermentation to produce an umami sauce and, for the first time, identified four novel umami peptides. This method both reduces pollution and yields a natural seasoning, thereby converting algal by-products into a valuable resource and offering the industry a sustainable “turning waste into treasure” model. Extending this approach, Zhu and co-workers (Contribution 7) used mackerel skin as feedstock and obtained highly active polypeptides via mixed-protease enzymatic hydrolysis; these peptides combine hair-repair and antioxidant functions, broadening the application of seafood by-products to functional personal-care products. Song and collaborators (Contribution 2) converted bullfrog skin into a multifunctional dual-network hydrogel that exhibits strong hemostatic and antibacterial performance, marking a successful transition from aquaculture waste to high-value biomedical material and further expanding the application scope of such by-products.

These contributions collectively highlight the modern seafood industry’s multi-dimensional development, encompassing full-chain innovations from processing optimization and safety risk prevention to by-product resource utilization. They emphasize the synergistic interaction among precise processing technologies, natural functional components, genomic analysis, and circular-economy thinking—all of which are directed toward the shared goal of producing safe, high-quality, and sustainable seafood. Together, they provide valuable technical guidance and theoretical support for researchers and industry practitioners.

## 3. Future Directions and Research Agenda

Despite recent advances in seafood quality and safety, novel processing methods and high-value uses for by-products, significant barriers remain to translating research into industry. Many studies on precision processing—such as vacuum freeze-drying and fermentation by native microbial consortia—and on functional component extraction are still confined to the laboratory. Their feasibility, economic viability and process stability at pilot and industrial scale therefore require urgent validation. A central challenge is achieving cost control and process efficiency without compromising nutritional and sensory quality, which is essential to drive industrial adoption of these technologies.

The quality management of seafood must be further developed from a full-chain perspective. The dynamic relationships among processing parameters, cold-chain logistics, microbial community evolution and consumer sensory preferences remain poorly characterized, which limits the applicability of current quality-assessment and risk-control systems. Future research should develop data-driven, multidimensional predictive models that integrate physicochemical indicators, microbial risk dynamics and storage environmental factors to enable proactive quality management and early warning across the supply chain.

The sustainability of seafood processing technologies must be supported by quantitative assessment. Life cycle assessment (LCA), together with technical and economic analysis, can objectively quantify energy use, water consumption and carbon emissions for emerging processing and sterilization technologies (such as cold plasma and conversion of enzymatic by-products), thereby informing decisions on green transformation. Equally important are safety compliance and consumer acceptance of novel products and techniques; these must meet international regulatory frameworks and the public’s expectations for natural, safe and sustainable marine food.

Intelligent technologies and interdisciplinary integration will increasingly drive innovation in the seafood industry. Process-optimization platforms that integrate hybrid intelligent sensing with digital twins are expected to enable real-time monitoring and adaptive control of processing operations. Concurrent advances across disciplines—such as marine-derived functional materials, edible coatings, and the integrated valorization of animal and plant by-products—will open new technological pathways for constructing a resource-circular, efficient, and resource-efficient marine food system. In sum, future seafood science will prioritize system integration and sustainable innovation, shifting the focus from laboratory-based research toward industrial-scale practice.

## 4. Conclusions

This Special Issue brings together recent advances in enhancing seafood quality, ensuring safety, and achieving the high-value utilization of by-products, highlighting the convergence of technological innovation and sustainable development. Collectively, the studies demonstrate that the future high-quality development of the seafood industry will not rely on a single technological breakthrough, but rather on collaborative innovation guided by systems thinking—optimizing quality through precise processing, ensuring safety via effective microbial risk management, improving resource efficiency through the high-value utilization of by-products, and embedding circular-economy principles throughout the entire value chain.

Although achieving a fully sustainable, resilient and high-quality seafood supply chain remains a long-term endeavor, the research presented in this Special Issue provides a robust scientific and technological foundation toward this goal. It is hoped that this Issue will stimulate further interdisciplinary collaboration and promote deeper integration across food science, microbiology, materials science, and data science, thereby accelerating the translation of laboratory findings into pilot-scale production and industrial applications. We anticipate that these continued efforts will sustain momentum toward the development of a safe, high-quality, environmentally responsible and sustainable seafood system.
